# The Use of Dietary Supplements to Alleviate Androgen Deprivation Therapy Side Effects during Prostate Cancer Treatment

**DOI:** 10.3390/nu6104491

**Published:** 2014-10-21

**Authors:** Andrea Dueregger, Isabel Heidegger, Philipp Ofer, Bernhard Perktold, Reinhold Ramoner, Helmut Klocker, Iris E. Eder

**Affiliations:** 1 Division of Experimental Urology, Department of Urology, Innsbruck Medical University, Innsbruck, A-6020 Austria; E-Mails: Andrea.Dueregger@student.i-med.ac.at (A.D.); Isabel-Maria.Heidegger@i-med.ac.at (I.H.); Philipp.Ofer@i-med.ac.at (P.O.); helmut.klocker@uki.at (H.K.); 2 Department of Dietetics, University of Applied Sciences Tyrol, Innsbruck A-6020, Austria; E-Mails: Bernhard.perktold@fhg-tirol.ac.at (B.P.); reinhold.ramoner@fhg-tirol.ac.at (R.R.)

**Keywords:** prostate cancer, androgen deprivation therapy, adverse effects, dietary supplements, alternative therapies

## Abstract

Prostate cancer (PCa), the most commonly diagnosed cancer and second leading cause of male cancer death in Western societies, is typically androgen-dependent, a characteristic that underlies the rationale of androgen deprivation therapy (ADT). Approximately 90% of patients initially respond to ADT strategies, however many experience side effects including hot flashes, cardiotoxicity, metabolic and musculoskeletal alterations. This review summarizes pre-clinical and clinical studies investigating the ability of dietary supplements to alleviate adverse effects arising from ADT. In particular, we focus on herbal compounds, phytoestrogens, selenium (Se), fatty acids (FA), calcium, and Vitamins D and E. Indeed, there is some evidence that calcium and Vitamin D can prevent the development of osteoporosis during ADT. On the other hand, caution should be taken with the antioxidants Se and Vitamin E until the basis underlying their respective association with type 2 diabetes mellitus and PCa tumor development has been clarified. However, many other promising supplements have not yet been subjected large-scale clinical trials making it difficult to assess their efficacy. Given the demographic trend of increased PCa diagnoses and dependence on ADT as a major therapeutic strategy, further studies are required to objectively evaluate these supplements as adjuvant for PCa patients receiving ADT.

## 1. Introduction

Prostate Cancer (PCa) is the most commonly diagnosed male cancer and the second leading cause of cancer death among men in Western societies [1,2]. Radical prostatectomy or primary radiation therapy are the preferred treatment modalities in men with locally confined PCa. For advanced tumors or tumor recurring after primary surgery or radiation therapy the androgen receptor (AR) and its signaling network are the prime targets of therapy. The androgen receptor orchestrates crucial oncogenic factors in PCa etiology since androgens drive proliferation, differentiation, and survival of benign and malignant prostate cells [3]. Hence, upon initial diagnosis, 80%–90% of PCa are androgen-dependent [4], an observation that underlies the rationale of androgen deprivation therapy (ADT), the current mainstay systemic treatment for advanced PCa [5]. Although highly effective, ADT is associated with considerable side effects that negatively affect the patient’s quality of life [6,7]. These adverse events include hot flashes [8], metabolic effects such as an induced metabolic syndrome (MetS) [9,10,11,12] including insulin resistance [13,14], cardiovascular (CV) diseases [15,16], musculoskeletal side effects characterized by reduced lean body mass and muscle strength, and osteoporosis [17,18,19,20] as well as depression and sexual dysfunction. Although several medical regimens have been developed [21], their impact on minimizing ADT side effects and improving quality of life is still under discussion. Recent statistics revealed an increasing use of complementary and alternative substances by PCa patients [22]. Indeed, approximately one in four patients with PCa uses at least one complementary or alternative method with the primary aim of ameliorating ADT-induced adverse effects [23,24]. In particular, herbal and dietary supplements appeal to patients because they are perceived as being “natural” with fewer side effects than prescription medicines. Despite the widespread use of alternatives to medical treatment options, little is known about their safety, efficacy and mechanism of action. The limitation of clinical studies investigating this issue leads to a lack of information concerning the use of different types of alternative interventions. This article focuses on the metabolic and musculoskeletal side effects of ADT, which are not alleviated by current treatment strategies. In particular, we discuss several adjuvant dietary options including herbs, phytoestrogens, selenium (Se), fatty acids (FA), calcium, and Vitamins D and E, whose use in the treatment of ADT side effects is supported by scientific evidence derived either from cell-based models, animal models or clinical trials.

## 2. ADT in the Treatment of PCa

**Figure 1:**
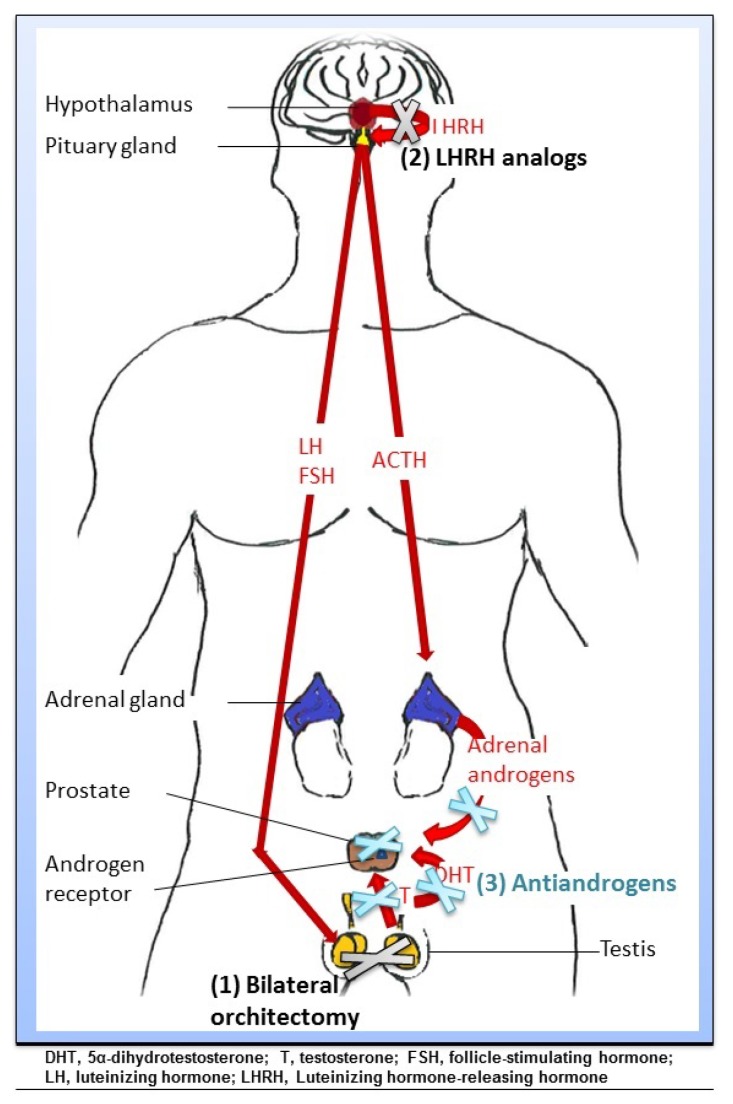
Mechanisms of action of androgen deprivation therapy (ADT) for blockage of the hypothalamus-pituary axis.

The androgenic hormones testosterone (T) and 5α-dihydrotestosterone (DHT), which mediate their action through the AR, are essential for normal prostate development but also contribute to prostate tumor growth by regulating cell proliferation and differentiation. The concept of hormonal manipulation using ADT to restrict PCa growth was first described in 1941 by Huggins and Hodges [25] and is based on the observation that 80%–90% of PCa are androgen dependent. Since then, multiple strategies have been established to reduce serum androgen levels or to interfere with their function by inhibiting the AR. Current strategies for hormonal blockade used in the treatment of PCa have been reviewed recently [26] and include bilateral orchiectomy, luteinizing hormone-releasing hormone (LHRH) agonists or antagonists and anti-androgens (Figure 1 and Table 1).

Current ADT strategies used in the treatment of PCa include bilateral orchiectomy (surgical castration), LHRH agonists or antagonists and anti-androgens (medical castration). (1) Bilateral orchiectomy is the surgical removal of both testicles inhibiting the production of testicular testosterone (T) and estradiol; (2) LHRH analogs reversibly decrease T production by the testis resulting in the down-regulation of LHRH receptors and thus reduced levels of luteinizing hormone (LH), follicle stimulating hormone (FSH) and T; (3) Anti-androgens (including flutamide, nilutamide, and bicalutamide) may bind competitively to the AR in cells thereby inhibiting its activation, or (abiraterone) block *de novo* androgen synthesis, both leading to apoptosis and reduced prostate tumor growth.

**Table 1:** Treatment options for hormone reduction.

Modality	Drug	Mechanism	Side Effects
**Surgical ** **Castration**			
**Bilateral Orchiectomy**	-	Surgical removal of testicles, ↓T	Hot flashes, reduced muscle mass and energy, anemia and osteoporosis
**Medical Castration**			
**LHRH Interference**	*LHRH agonists* Leuproreline acetate (Trenantone^®^, Eligard^®^) Goserelin (Zoladex^®^) Triptorelin (Trelstar^®^) Histrelin (Vantas^®^)	LHRH receptor downregulation after initial flare, ↓LH, ↓FSH, ↓T	Hot flashes, reduced muscle mass and energy, anemia and osteoporosis, flare phenomenon, CV events, cardiotoxicity
	*LHRH antagonists* Degarelix (Firmagon^®^)	Blockade of LHRH receptor, ↓LH, ↓FSH, ↓T	Hot flashes, reduced muscle mass and energy, anemia and osteoporosis, CV events, histamine release
**Antiandrogens**	*Non-Steroidal* Flutamide (Eulexin^®^) Bicalutamide (Casodex^®^) Nilutamide (Nilandron^®^) Enzalutamide (Xtandi^®^)	Antagonizes AR in target tissues, ↑T	Gynecomastia, hepatotoxicity (flutamide), visual and respiratory disturbance and alcohol intolerance (nilutamide), GI problems ,fatigue and hot flushes (enzalutamide)
	*Steroidal* Cyproterone acetate (Androcur^®^, Cyprostat^®^)	Antagonizes AR in target tissues, suppress LHRH secretion, ↓LH, ↓T	Gynecomastia, cardiovascular events, fluid retention, GI problems
	Abiraterone acetate (Zytiga^®^)	Inhibition of Cyp17A1 enzyme, suppresses T, estrogen and glucocorticoid biosynthesis	Vomiting, GI problems, swellings, weakness, cough, high blood pressure

LHRH, luteinizing hormone-releasing hormone; LH, luteinizing hormone; T, testosterone; AR, androgen receptor; GI, gastrointestinal; CV, cardiovascular.

Although accompanied by fewer side effects than “medical castration”, the use of surgical bilateral orchiectomy is currently limited in developed countries. Rather, medical-based approaches to achieve castration levels of circulating androgens are preferred. In contrast to surgical castration, medical castration using LHRH analogs reversibly decrease T production by the testis and are therefore the preferred treatment modality. There are two different classes of LHRH analogs: LHRH agonists and antagonists. LHRH agonists stimulate the LHRH receptors in the pituitary gland resulting in a temporary increase in LH and FSH secretion, which in turn causes an initial rise in T production (the so-called “flare phenomenon [27]”). However, constant LHRH stimulation leads to a negative feedback loop resulting in the down-regulation of LHRH receptors and thus reduced levels of LH, FSH and T. In contrast to LHRH agonists, LHRH antagonists competitively bind to their receptors in the pituitary gland thereby blocking their activation by the natural ligand, inducing a rapid decrease in LH, FSH, and T levels without any flare.

Anti-androgens differ mechanistically from the above-described castration therapies as they do not alter androgen induction by direct modification of the hypothalamic-pituitary-gonadal axis in the brain. Rather, most anti-androgens (including flutamide, nilutamide, and bicalutamide) bind competitively to the AR in cells inhibiting its activation, leading to apoptosis and reduced prostate tumor growth. By contrast, abiraterone blocks *de novo* androgen synthesis via irreversible inhibition of CYP17-A1, a rate-limiting enzyme that catalyzes the conversion of cholesterol to androgen and estrogen precursors. The new-generation drugs abiraterone and enzalutamide have been developed during the past 5 years [28] and thus knowledge concerning their side effects is still limited. Since serum T and estrogen levels are maintained for all anti-androgen drugs that target the AR, hypogonadal side effects are generally less pronounced [29]. However, anti-androgens are often used in combination with LHRH analogs, thus this article will not discuss the side effect profile of anti-androgen monotherapy.

## 3. Adverse Effects of ADT

Frequent side effects of ADT that result in poor quality of life include hot flashes, metabolic effects such as gynacomastia as well as an increased body mass index, insulin resistance, metabolic syndrome (MetS), cardiovascular (CV) diseases, and musculoskeletal effects including reduced muscle mass, osteoporosis, and also sexual dysfunction (summarized Table 2) [1,7,30,31,32,33,34,35] Of these adverse events, metabolic and musculoskeletal effects are the most prevalent and distressing side effects reported by patients [21].

**Figure 2:**
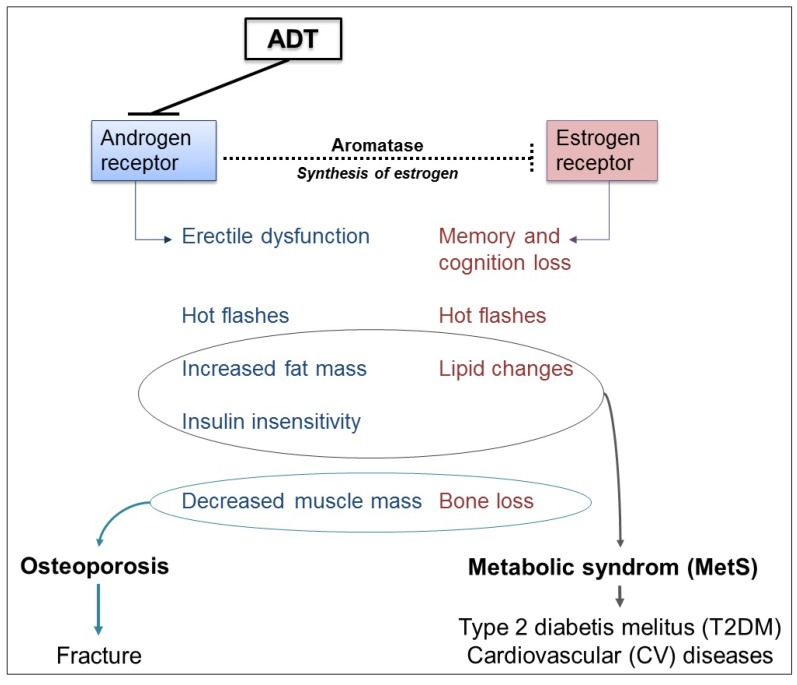
Side effects associated with androgen deprivation therapy (ADT).

ADT targets gonadal function. Consequently, hypogonadism is prevalent in PCa patients undergoing ADT compared to those that undergo surgery and/or radiation therapy or compared to age-matched controls [7]. Thus, ADT induces a profound hypogonadism, which in turn is responsible for increased body mass index, increased fat mass, reduced lean body mass and muscle strength, and osteoporosis. Besides the desired physiological consequences of ADT in reducing serum androgens, hormonal castration is also associated with a decrease in circulating estrogens that are synthesized from androgens by peripheral aromatization (Figure 2). Despite having normal to elevated serum T levels, men with congenital aromatase deficiency (and thus, non-detectable serum estrogen levels) have a high prevalence of osteoporosis, insulin resistance and metabolic syndrome (MetS) [36], an observation that underscores the importance of estrogens in men. Moreover, it is decreased estrogen rather than T levels that are responsible for decreased bone density, accelerated rate of bone loss, and increased fracture incidence [37]. Thus, side effects induced by ADT leading to hot flashes, osteoporosis, MetS and higher CV events are related to androgen as well as estrogen deficiencies [35,38]. However, the relative contribution of T and estrogen to these adverse effects remains unclear.

ADT is associated with multiple adverse effects, many of which are related to androgen as well as estrogen deficiency that occur as a result of treatment [30,35].

**Table 2:** Summary of dietary supplements for the management of androgen deprivation therapy (ADT) side effects.

Side Effect	Postulated Management	Efficacy	Reference
**Hot Flushes**	**Herbs**		
	*Black cohosh*	Minimal, ↓sweating symptoms, may ↓hot flashes in women.	[39,40]
	*Dong quai*	No benefit in women or men.	[41]
	*Ginseng*	Minimal effects in women.	[42]
	**Phyto-Estrogens**		
	*Soy-Isoflavons*	No effect with supplements in men; possible impact with dietary source seen in women.	[43,44,45,46,47,48,49]
	*Flaxseed*	Unknown, initial studies showed impact on hormonal levels and serum lipids.	[50]
	**Vitamin E**	Previously recommended, but increased risk for diabetes. Might also increase the risk for PCa (SELECT trial).	[43,51,52,53,54,55,56,57,58]
**Osteoporosis**	**Herbs**		
	*Black cohosh*	Potentially effective in preclinical studies (only studied in female animals).	[40,59]
	**Phyto-estrogens**		
	*Soy-Isoflavones*	Potentially effective in preclinical studies (castrated male animals).	[60,61]
	**Fatty acids**		
	*Omega-3 FA* *(CLA* *)*	Potentially effective in preclinical studies (male and female animals).	[62,63,64,65,66]
	**Calcium and** **Vitamin D**	Effective in men and postmenopausal women.	[67,68,69,70,71,72]
**Cardiovascular Events**	**Herbs**		
	*Ginseng*	No benefit.	[42]
	*Garlic*	Showed to reduce blood pressure.	[73,74]
	**Phyto-Estrogens**		
	*Soy-Isoflavones*	No effect. Might reduce ↓LDL-cholesterol.	[46,75,76]
	*Flaxseed*	Potentially effective, reduced ↓LDL-cholesterol in postmenopausal women.	[77]
	Fatty acids		
	*Omega-3 FA*	No benefit. Postulated mechanism: ↓TG	[78,79,80,81,82]
	**Selenium**	No effect. Might have adverse effects on diabetes. Increased PCa risk (SELECT trial).	[24,56,58, 83,^84^,^85^,^86^,^87^,^88^,^89^,^90^]
	**Vitamin E**	Negative association with CV health. Might increase PCa risk. No benefit in women.	[43,51,55, 57,^58^,^86^, 91,^92^]

CV, cardiovascular; CLA, conjugated linoleic acid; LDL, low density lipoprotein; TG, triglyceride.

### 3.1. Metabolic and Cardiovascular Side Effects

A number of prospective studies have shown that ADT increases abdominal fat and serum triglycerides (TG) and decreases insulin sensitivity [93]. Additionally, cross-sectional studies have shown that men receiving ADT are more likely to meet the diagnostic criteria of MetS [94,95]. Moreover, long term ADT therapy caused significantly higher levels of fasting insulin and glucose compared to men with PCa not on ADT as well as to age-matched controls [96]. A retrospective study involving 44 PCa patients who received ADT showed an increase in fasting blood glucose, total cholesterol, LDL cholesterol, and TG, but a decrease in HDL cholesterol in these patients [34]. In addition, longitudinal studies indicate that lower T levels in men independently predict MetS [97,98] and type 2 diabetes mellitus (T2DM) [99]. Because MetS is independently associated with CV mortality [100], it is plausible that the positive association between lower serum T levels and MetS may at least in part explain the higher CV mortality in men with PCa receiving ADT (with therapy-induced hypogonadism as the likely trigger of these events) [101]. The protective role of T on the CV system and its relationship to MetS, CV morbidity and mortality has been recently reviewed [98]. Since CV disease has become the most common cause of non-PCa-related death among this patient group [102], the potential risks of ADT with regards to CV events should be carefully weighed against the expected benefits. Moreover, since metabolic complications predominantly occur within 3–6 months after starting ADT therapy, close observation of patients especially during the first year of ADT is highly recommended [93].

### 3.2. Musculoskeletal Side Effects

Given that 25–40% of patients already have osteoporosis at PCa diagnosis, it must be considered that the symptoms will worsen upon administration of ADT. In general, two different forms of osteoporosis are known [103]. Whereas primary osteoporosis is caused by malnutrition and genetic predisposition, secondary osteoporosis predominantly arises in men due to hypogonadism, including that experienced by PCa patients receiving ADT [104]. Longitudinal studies report that approximately 35% of men on LHRH agonists experience at least one skeletal fracture and approximately 20% will be diagnosed as osteoporotic or osteopenic within 7 years of starting therapy [105]. The absolute excess risk of fractures ranges from 5%–7% over an average of 6 years follow-up compared to men not receiving ADT [31,106]. In particular, treatment with either LHRH analogs or orchiectomy is associated with a significant decline in bone mineral density (BMD) and increased risk of bone fractures [31,106]. In this context, it should be noted that bone fractures in men with PCa have been associated with higher mortality rates [107]. Cross-sectional studies have shown that BMD at the hip and lumbar spine decreases with the duration of continuous ADT [108]. However, BMD appears to initially decline at a fast rate. For example, greater declines of BMD were observed among recent *versus* chronic ADT users with stronger declines in both groups in contrast to ADT naïve men [109]. BMD declined at multiple sites including the lumbar spine and hip in the first year after starting ADT, however subsequent changes in the second year were much smaller and no longer statistically significant [110]. A larger study followed 618 men on ADT for up to 7 years with annual dual-energy X-ray absorptiometry (DXA) scans [111]. Whilst steady annual declines in BMD among men with normal BMD at baseline were noted, only 38 men with normal BMD at baseline still remained in the study by 3 years [111]. Thus, due to the lack of longitudinal studies, it is currently not possible to determine whether BMD loss beyond the first year of ADT is attenuated.

## 4. Dietary Supplements

The use of dietary supplements to alleviate side effects of ADT is an attractive approach for the majority of PCa patients. Indeed, an array of dietary supplements proposed to be useful in intervening with side effects due to declining hormone levels in men and women are promoted typically via media that lack scientific evidence. However, it should be noted that many of these supplements have been studied in the context of hormone deprivation during menopause or in the course of breast cancer therapy in women with no clinical studies carried out in men thus far (e.g., black cohosh, ginseng, garlic). Thus, it is difficult to conclude whether these dietary supplements will exhibit a similar efficacy in PCa patients receiving ADT. Of the few dietary supplement studies that have been performed in men, most were primarily aimed at reducing the risk of PCa development or reducing the risk of disease progression after initial diagnosis or first line treatment. Thus, studies in men receiving ADT are rare. Consequently, it is inappropriate to assume that beneficial (or non-beneficial) effects on PCa development or progression will similarly translate to modifying the adverse effects associated with ADT.

### 4.1. Herbs

Herbal supplemental approaches to manage metabolic and musculoskeletal side effects like hot flashes and osteoporosis have been extensively evaluated in breast cancer and menopausal women in small randomized trials with varying success [112,113]. Thus, it seems evident to evaluate the potential of the most promising of these herbs for the treatment of side effects in men who are being treated for PCa with ADT.

#### 4.1.1. Black Cohosh (*Cimicifuga racemosa*)

The herbal supplement black cohosh, which is approved for the treatment of menopausal symptoms by the German health authority (Commission E), has also shown to have MetS-preventive and bone-protective properties in recent preclinical studies [59,114]. A randomized, placebo-controlled trial found no difference in the frequency of hot flashes but a significantly lower incidence of sweating in menopausal women with a history of breast cancer in the black cohosh group compared to placebo [39]. This finding is particularly pertinent given that a significant placebo effect is consistently observed in investigations of hot flashes, with the placebo effect reportedly sufficient to reduce hot flashes by up to 75% [115]. On the other hand, significant evidence of potentially beneficial effects on bone tissue was shown, although it should be noted that all clinical studies were conducted in females [59,116]. Of interest is one study that was carried out in orchidectomized male rats and showed to prevent osteoporosis *in vivo* [117].

The mechanism underlying the activity of black cohosh remains poorly understood. There is a lack of scientific evidence regarding its proposed estrogenic activity. However, it was recently shown to attenuate nucleoside uptake into cells and thus may have an impact on tumor treatment by nucleoside analogs [118]. One may speculate that its modulation of adenosine signaling may be beneficial for CV side effects of ADT [119], but this has yet to be investigated. Despite the lack of knowledge regarding the mechanism of action of black cohosh, its long history of use reveals that black cohosh is well tolerated. The use of black cohosh in PCa patients undergoing ADT may be warranted for hot flushes, CV and osteoporotic side effects. Importantly, further systematic studies assessing the safety and efficacy of black cohosh in alleviating ADT-induced side effects may be worth pursuing.

#### 4.1.2. Dong Quai (*Angelica* *sinensis*)

Dong quai is a traditional Chinese herbal remedy most commonly used in the treatment of female reproductive problems. According to our knowledge, there are no pre-clinical studies addressing its effects in PCa. However, a small randomized clinical trial was conducted in men receiving ADT where dong quai was shown to be ineffective in reducing hot flashes [41]. Similarly, randomized trials in women also found no effect of dong quai on hot flashes beyond a placebo, irrespective of whether the herb was used alone or as part of a complex multi-ingredient intervention [120]. Taken together, the current evidence does not support the use of dong quai in patients undergoing ADT.

#### 4.1.3. Ginseng (*Panax ginseng*)

Ginseng extract is widely used in traditional Chinese medicine and was reported to reduce fatigue, insomnia and depression in post-menopausal women, although there was no significant benefit on hot flashes [121]. However, a recent review of studies examining the efficacy of ginseng on menopausal symptoms highlighted the poor quality and bias of many randomized clinical trials conducted to date, raising doubt as to the usefulness of this herb in managing menopause symptoms [42]. Nonetheless, several pre-clinical and clinical studies have shown that ginseng may possess anti-cancer and hypoglycemic properties, the latter of potential benefit in ameliorating MetS in men receiving ADT [122,123].

#### 4.1.4. Garlic (*Allium sativum*)

Garlic is frequently used as a dietary supplement for the treatment of hyperlipidemia, heart disease, and hypertension [124]. In addition, there is evidence that garlic is associated with blood pressure reduction in patients with elevated systolic blood pressure (10–12 mmHg systolic, 6–9 mmHg diastolic) but not in normotensive patients [73,74]. In this respect, it is conceivable that garlic may also reduce CV effects in PCa patients undergoing ADT. However, there is currently insufficient data to support this hypothesis and further studies that specifically address this question would be required [125].

### 4.2. Phytoestrogens

In the past, suppression of T was achieved using high doses of estrogens (estradiol) or selective estrogen receptor modulators [126,127,128,129]. However, these treatments were prone to severe and even fatal CV side effects [130,131]. Consequently, these treatments have been replaced by other therapeutics such as LHRH analogs. Phytoestrogens (plant estrogens) are non-steroidal naturally occurring phenolic compounds with known estrogenic effects and estrogen receptor (ER-β and/or ERα) binding properties [132,133,134]. Thus, these “mild” estrogens could possibly serve as natural alternatives with potentially fewer side effects. Indeed, phytoestrogens have been shown to improve metabolic health, reduce CV risk, and improve BMD and brain function [60,75,135]. There are three classes of phyto-estrogens, which are categorized according to their chemical structure as isoflavones, lignans or coumestans. Isoflavones are the largest and also the most extensively studied group of phytoestrogens, which includes genistein, daidzein and glycitein. Isoflavones are found in highest amounts in soybeans, flaxseed and legumes. Soy is stably integrated into Asian diets where daily intake is at least 40 times higher than among Western populations [136]. This is considered to be one of the factors contributing to a much lower incidence of prostate and breast cancer in Asian countries [137]. In addition, increased consumption of isoflavones has been associated with decreased incidence of diabetes and heart disease in South and East Asia [138,44]. In clinical trials, isoflavones have shown an improvement in hot flash severity, glycemic control, MetS and inflammatory profile in both men and postmenopausal women [45,76,139]. However, in a recent double-blinded, randomized, placebo-controlled pilot study, phytoestrogens failed to improve metabolic or inflammatory parameters of men with PCa during ADT [47]. This is consistent with another pilot study conducted in 33 men where high dose isoflavones showed no significant improvement in cognition, vasomotor symptoms or any other aspect of quality of life measures compared to placebo in androgen deprived men [140]. Thus, there is currently no clinical evidence for the proposed improvement of ADT-induced side effects by phytoestrogens, although limitations of these studies including small cohort sizes and short treatment durations should be taken into account.

### 4.3. Selenium (Se)

Se is an essential trace element, which is incorporated as selenocysteine into selenoproteins, many of which are reactive oxygen species (ROS) scavenging enzymes, such as glutathione peroxidase and thioredoxin reductase [141,142]. Thus, Se may be useful in decreasing oxidative stress and low-grade inflammation. Notably, plasma biomarkers of oxidative stress and low-grade inflammation are associated with MetS, obesity and insulin resistance, conditions that are common side effects of ADT [143]. Se was shown to have strong anti-proliferative and pro-apoptotic effects on human PCa cells and to improve severe metabolic side effects in patients [144,83]. Moreover, cancer prevention studies indicated that Se decreases ROS and is associated with decreased incidence of PCa [145,146]. Taken together, these observations provided a strong mechanistic rationale to combine ADT and Se for the treatment of PCa [5] and led to a number of epidemiological studies and clinical trials [83,51]. However, epidemiological studies have suggested that supranutritional Se intake and high plasma Se levels are not necessarily preventive against cancer, and may even be a possible risk factor for developing T2DM [83]. For example, supplementation with Se and/or Vitamin E in the large-scale Selenium and Vitamin E Cancer Prevention Trial (SELECT) did not prevent the development of PCa, rather, the incidence of newly diagnosed T2DM increased among the Se-supplemented participants [83,51]. Whilst the Nutritional Prevention of Cancer (NPC) study reported a decreased risk of PCa among Se supplemented men, an increased risk of T2DM was also observed in participants with baseline plasma Se levels in the top tertile [147]. Since then, several longitudinal studies have failed to support a causal role of Se in T2DM, although cross-sectional studies continued to find significant associations between circulating Se levels and T2DM [148]. For example, serum Se was observed to be associated with adipocytokines, such as TNF-α, VCAM-1, leptin, FABP-4, and MCP-1 [149,150] and adiponectin [151]. Although on the other hand, an analysis across randomized groups showed that Se supplementation had no effect on adiponectin levels after six months of treatment [84]. Thus, it appears likely that the reported link between Se and T2DM is due to indirect effects of Se-containing ROS scavenging enzymes, affecting the hydrogen peroxide level that in turn modulates both glucose-induced insulin secretion and insulin-induced signaling [152,153]. Moreover, Se homeostasis is modulated by factors related to carbohydrate metabolism, suggesting that low serum Se levels may themselves be a consequence of dysregulated energy metabolism in T2DM [153]. In addition, there appears to be a significant interaction between dietary intake of phytoestrogens and Se with important implications for heart disease, cancer, diabetes, and other conditions related to body weight [154].

From the current prospective—especially since to date there has been no study specifically evaluating the supplementation of Se in PCa patients receiving ADT—a combination of ADT with Se cannot be recommended given that many patients develop pre-diabetes.

### 4.4. Fatty Acids (FA)

The relative amounts and different types of dietary FA are thought to play critical roles in PCa associated MetS. Total FA intake and the ratio of omega-3 (ω-3) to omega-6 (ω-6) polyunsaturated FA (PUFAs) in the Western diet have increased significantly since the Industrial Revolution [155]. The effects of ω-3 PUFAs on CV disease, cancer as well as MetS have been investigated extensively [78,156,157,158,159,160,161]. Consequently, the United States Food and Drug Administration (FDA) has approved ω-3 PUFAs for the prevention of CV adverse outcomes by the postulated mechanism of lowering serum triglyceride levels [162,163]. Additionally, three common dietary ω-3 FA—alpha-linolenic acid (ALA), eicosa-pentaenoic acid (EPA), and docosahexaenoic acid (DHA)—were proposed to exhibit anti-inflammatory properties [50,79,164]. Since inflammation is a major risk factor for the development of CV disease, these ω-3 FA may reduce the risk of CV disease [50,79,164]. Various pre-clinical, epidemiological and clinical studies have investigated the influence of ω-3 and ω-6 FA on the development and progression of PCa [80,155,158,161,165,166,167,168,169,170,171,172,173]. However, these studies have yielded contradictory results. In particular, dietary intake of long-chain ω-3 PUFA or its individual components (EPA, DHA, docosapentaenoic acid (DPA) and ALA) have been associated with PCa risk and progression [80,155,158,161,165,166,167,168,169,170,171,172,173]. On the other hand, encouraging results were obtained from clinical trials showing potential anti-inflammatory effects of ω-3 FA [77,174,175,176,177]. Other trials investigating higher doses of ω-3 FA (>1 g/day EPA and/or DHA) in populations at high risk for CV disease reported improvements (*i.e.*, reduced concentrations) of selected inflammatory markers [81,178,179]. However, it should be noted that in these studies the same dosage of ω-3 FA showed mixed response rates in healthy adults, and no beneficial effect in patients that received lower doses (<1 g/day) [81,174,175,178,179,180,181,182,183,184,185]. A single study has reported that very high intakes (6.6 g/day), which are well beyond the current recommendations (500 mg/day–1 g/day), may raise blood concentrations of some inflammation markers such as soluble tumor necrosis factor receptors 1 and 2 (sTNF-Rs 1 and 2) [79,185]. It is likely that differences in dosage, study population characteristics, the source of ω-3 FA, study duration, and background diet may explain the inconsistencies in these findings. In this respect, it may be noted that the majority of clinical trials have used marine sources of ω-3 FA (EPA and DHA from fish), whereas few have examined FA from plants (ALA from flax oil) and only two trials compared both sources [175,82]. The heterogeneous results from these studies might also be due to variations in measuring FA consumption of individuals and different techniques assessing their diet [186,187,188,189,190]. The main mechanisms underlying the purported anticancer effects of modulating dietary fat appear to be through reduced insulin-like growth factor signaling and alterations in membrane ω-6 to ω-3 FA ratios leading to suppressed COX-2-dependent PGE-2 production and reduction of inflammation via modification of the eicosanoid pathway [191,192,193,194,195]. Moreover, decreased PGE-2 levels are expected to decrease estrogen production and further also modify androgen production [190].

In summary, although ω-3 FA exhibits some anti-inflammatory potential, there is still a lack of consensus regarding their optimal use and dosage. Moreover, results from the aforementioned studies have to be interpreted with caution, since the metabolic conditions during ADT are different. Further research is warranted to better elucidate the mechanism of action and ideal consumption of ω-3 FA for potential CV health benefits during ADT. 

### 4.5. Calcium and Vitamin D

Osteoporosis is a common and one of the most debilitating side effects of ADT. The most important nutritional factors contributing to osteoporosis include deficiencies in Vitamin D and calcium. Measurement of Vitamin D levels in osteoporotic males by a large multi-center study in the US (MrOs) revealed a deficiency in 26% and an insufficiency in 72% of subjects [54]. Because deterioration of BMD occurs soon after initiation of ADT therapy [67], the European Association of Urology does not specify recommendations but states that calcium supplementation is protective [196]. The National Comprehensive Cancer Network cite the National Osteoporosis foundation guidelines that recommend calcium (1000 mg daily) and Vitamin D (800–1000 IU daily) supplementation for all men aged 50 or above [197]. Notably however, data supporting this recommendation are lacking as shown in a recent systematic review [196]. One cross-sectional study suggested an association between low calcium intake and a greater likelihood of osteoporosis in men with PCa of whom 71 % were undergoing ADT. Unfortunately, Vitamin D use was not examined in this study [198]. Another trial reported a positive association of calcium and Vitamin D use (examined together) on hip and lumbar spine BMD in men on ADT [199]. Importantly, results from 12 different clinical trials revealed that the commonly recommended doses, of 500–1000 mg calcium and 200–500 IU Vitamin D per day still result in BMD loss in men receiving ADT [196]. Alibhai *et al.* examined long-term effects of calcium and Vitamin D in a prospective 3-year matched cohort study comparing PCa patients with and without ADT. This study found that Vitamin D but not calcium may be protective particularly in the first year of ADT [32]. In a multivariate analysis, it was further shown that the mean daily calcium intake in men receiving ADT was significantly lower in men who suffered from osteoporosis compared to those without osteoporosis [198].

In summary, calcium and Vitamin D supplementation is a recommended complementary therapy not only in elderly men with osteoporosis but also in men undergoing ADT even though the long-term impact of ADT on BMD and the value of calcium and Vitamin D in ameliorating negative effects remains to be elucidated more precisely.

### 4.6. Vitamin E

Vitamin E is a potent antioxidant, which is of interest to ameliorate hot flushes and CV associated side effects of ADT. Vitamin E has a long history in the treatment of pre-eclampsia (characterized by high blood pressure in pregnant women), premenstrual syndrome, painful periods, menopausal syndrome, hot flashes associated with breast cancer, and breast cysts even though randomized controlled clinical studies did not reveal any benefit [52,53,86,200]. Moreover, the Physicians Health Study II concluded that Vitamin E does not reduce the risk of major CV events (non-fatal myocardial infarction, non-fatal stroke, or CV disease death) [57]. Similarly, the Women’s Health Study, which comprised approximately 40,000 healthy women, found that Vitamin E did not reduce the risk of death or major CV events. Interestingly however, there was a significant reduction in the secondary endpoint of CV deaths and in major CV events among a subgroup of women aged 65 or over [55]. The Women’s Antioxidant Cardiovascular Study found that there were no overall effects of Vitamin E on CV events among women at high risk of CV disease [91]. Moreover, the intake of Vitamin E has been shown to increase all-cause mortality and may even have negative effects on CV health [92]. 

There have been a number of studies in men, which have purported a positive effect of Vitamin E on hot flushes and high blood pressure [201]. However, most of these studies have yielded inconclusive or conflicting findings or a lack of benefit for its use, illustrating the need for studies of higher quality in this area. Thus, clinical trials have failed to recapitulate the promising findings of *in vitro* and many observational studies. Possible reasons for this discrepancy may be that clinical trials are too short in duration to reverse the results of decades of oxidative stress contributing to atherosclerosis or that the antioxidants selected for study were chosen for their ease of availability rather than proven efficacy (Vitamin E) [202]. Recent evidence from the SELECT trial revealed an increased risk for PCa in the Vitamin E supplemented group. Taken together, current evidence does not support a beneficial effect for Vitamin E, and its use as a supplementary treatment is therefore not recommended [56,88].

## 5. Discussion

The administration of ADT is associated with a diverse set of known side effects that have a significant impact on patient quality of life, overall health, and mortality. Some of the dietary supplements discussed in this review may be beneficial for patients undergoing ADT. The value of calcium and Vitamin D supplementation remains to be elucidated more precisely; however, because of their long and safe history of usage they may be recommended in the prevention of osteoporosis during ADT. Phytoestrogens were shown to prevent hot flashes and other climacteric complaints and exert anti-osteoporotic effects in women. However, positive effects on CV health are still questionable and require further elucidation, especially with respect to their effects in PCa patients receiving ADT. In addition, further clinical trials evaluating the efficacy of isoflavones must be conducted before their use for relieving ADT-induced side effects can be advocated. We can conclude that dietary interventions with herbal substances may in fact be helpful in the treatment of adverse effects arising from ADT [43]; however, more clinical randomized studies in PCa patients on ADT are highly warranted to support these findings. The long history of use and lack of adverse effects of black cohosh in the treatment of climacteric complaints in women is particularly encouraging. Further evaluation of its proposed osteo-protective and anti-metabolic effects in conjunction with ADT in randomized and controlled clinical studies is also warranted. Recent data obtained in the large SELECT trial suggest that combined supplementation of Vitamin E and supranutritional Se may increase the risk of PCa, making this a non-recommended treatment for men receiving ADT. From the current perspective, a combination of ADT with Se, which has been associated with an increased risk for the development of T2DM, cannot be recommended given that many patients develop pre-diabetes.

## 6. Conclusions

In summary, dietary supplements are active compounds with as yet mostly poorly defined effects. As such, the unregulated self-prescription of active compounds such as soy, Se or Vitamin E may in some cases even prove to be harmful or negatively interfere with cancer treatment. Given the prevalent use of alternative dietary supplements in PCa patients, there is an urgent need to (1) perform rigorous research to determine the precise physiological effects of these different supplements with respect to relieving side effects of ADT; (2) conduct clinical trials of these supplements in men undergoing ADT and (3) establish more open lines of communication between patients and physicians regarding the use of dietary supplements and their integration into conventional treatment strategies.
